# Time for trauma immunology

**DOI:** 10.1371/journal.pmed.1002342

**Published:** 2017-07-11

**Authors:** Timothy R. Billiar, Yoram Vodovotz

**Affiliations:** Department of Surgery, University of Pittsburgh Medical Center, Pittsburgh, Pennsylvania, United States of America

## Abstract

Timothy Billiar summarizes the role of the innate immune response in the clinical course following severe injury and envisions a field of "trauma immunology."

While it is intuitive that the damage to bones and soft tissues causes both short- and long-term “organic” dysfunction, the “illness” caused by injury is less appreciated. This results from the systemic manifestations that follow the physiologic, immunologic, and metabolic changes induced by shock from blood loss and direct tissue destruction. The immunologic changes following injury are profound, and have been measured in the circulating leukocytes of injured humans as a massive activation of the innate immune system and a near-simultaneous impairment in adaptive immune responses [1]. This “immune dysfunction,” seen after even moderate injury, can be appreciated clinically as inflammation-associated organ dysfunction (typically peaking on day 2 to day 3 postinjury) and a sustained increase in the susceptibility to secondary infections, especially pneumonia [1,2]. The 2 processes are thought to be linked by the proximal events that activate the immune system immediately following a traumatic event.

## PAMP/DAMP paradigm

The quest to understand how the immune system becomes activated by trauma lags considerably behind, and is ultimately informed by, discoveries regarding mechanisms of microbial recognition by immune cells. That immune recognition systems are gene-encoded in mammals was predicted by studies in the 1960s, showing that certain strains of mice are resistant to bacterial endotoxin [3]. By 1993, the parallels between microbial immune recognition and injury recognition were drawn closer when DeMaria and colleagues showed that the endotoxin-resistant C3H/HeJ mouse strain was also resistant to hemorrhagic shock-induced death [4]. The discovery that the endotoxin-resistant mouse strain possessed a mutation in the Toll-like receptor (TLR)4 gene, the mammalian homologue to the pattern-recognition receptor first described in drosophila [5], subsequently confirmed the prediction that the innate recognition of microbial (nonself) antigens by the host would involve gene-encoded receptors [6]. Of course, we now know that several families of pattern-recognition receptors, including TLRs, Nod-like receptors (NLRs), C-type lectin receptors (CLRs), and retinoic-acid–inducible protein 1 (RIG-I)-like receptors, continuously survey the extra- and intracellular compartments for molecules expressed by foreign invaders. These molecules are referred to as pathogen-associated molecular patterns (PAMP).

It was soon realized that a subset of these receptors could also recognize host-derived molecules and that this recognition could lead to robust immune-cell activation and production of inflammatory mediators, following insults common to severely injured humans—hemorrhagic shock and tissue damage [7,8]. This realization provided support for the concept that the immune system recognizes not just nonself but any threat to host homeostasis—the so-called “danger theory” [9]. Self-derived molecules that trigger pattern-recognition receptor signaling are referred to as damage-associated molecular pattern (DAMP) molecules and encompass both cellular (e.g., high-mobility group box 1, mitochondrial DNA) and matrix components (e.g., hyaluronan). Evidence continues to accumulate to support a DAMP “surge” postinjury as the seminal event in trauma-induced immune activation [2,10]. This release may be an active process induced by tissue hypoxia resulting from hypo-perfusion, or passive as the result of cell lysis. Persistent or repeated cycles of DAMP release may heighten or maintain the overactivation of the immune system during the escalation phase that characterizes the path to immune dysfunction, essentially forming a forward feedback loop of tissue damage leading to inflammation that in turn leads to further tissue damage and cellular dysfunction.

The immune dysfunction process following trauma is dominated by the pattern-recognition receptors TLR2, TLR4, and TLR9, although roles for other pattern-recognition receptors are likely. To date, TLR4 appears to be the most promiscuous DAMP-responsive receptor, recognizing a wide array of endogenous molecules independent of gram-negative bacterial endotoxin [7]. TLR9 is also particularly interesting as a DNA receptor, which may be linked to the immune activation resulting from the release of mitochondrial DNA [8,10]. Mitochondria may be an especially important source of DAMP molecules, based not only on their proposed bacterial origins but also because mitochondrial DNA and other mitochondrial molecules are elevated in trauma patients that progress to critical illness [10].

It is tempting to refer to trauma-induced immune activation as “sterile inflammation”; however, it is just as likely that the propagation of the immune response involves PAMP molecules derived from the host’s own microbiome or microbes invading traumatized tissues. This interplay between DAMP and PAMP signaling remains a fruitful area for additional investigation.

## Trauma immunology

The immune response associated with trauma-induced “critical illness” is characterized by both its magnitude and unique inflammatory mediator patterns [1,2]. Other major health conditions associated with aberrant immune regulation have led to highly specialized areas within immunology, for example, cancer (tumor immunology), cell and organ transplantation (transplant immunology), and the broad number of diseases resulting from abnormal recognition of self by the immune system (autoimmunity). The creation of clearly defined fields of study within immunology has contributed in the translation of basic discoveries into new therapies. Based on the unique dynamics of the immune response to trauma, the scope of the clinical problem (third overall cause of death), and the tremendous opportunity for targeting the immune system in appropriate patients, the rationale appears to exist to define a new field within immunology: “trauma immunology.” The goal would be to bring trauma under the umbrella of mainstream immunology and build upon the recent advances that have begun to link early postinjury events to immune pathways previously thought to be activated only in chronic, autoimmune, or inflammatory processes [11]. Given the likely need to integrate preexisting host factors such as genetic variability and microbiome composition with DAMP and PAMP signaling, this nascent field is poised to benefit from major strides in systems and computational immunology [12]. Likewise, insights derived from the study of the immunology of traumatic events (whose origin in time can be mapped precisely to the moment of injury) will aid in understanding chronic inflammation and immune disorders in the larger context.

## Abbreviations

CLR: C-type lectin receptor
DAMP: damage-associated molecular pattern
NLR: Nod-like receptor
PAMP: pathogen-associated molecular pathways
RIG-I: retinoic-acid–inducible protein I
TLR: Toll-like receptor
